# The material-weight illusion disappears or inverts in objects made of two materials

**DOI:** 10.1152/jn.00199.2018

**Published:** 2019-01-23

**Authors:** Vivian C. Paulun, Gavin Buckingham, Melvyn A. Goodale, Roland W. Fleming

**Affiliations:** ^1^Department of Psychology, University of Giessen, Giessen, Germany; ^2^Brain and Mind Institute, Western University, London, Ontario, Canada; ^3^Department of Sport and Health Sciences, College of Life and Environmental Sciences, University of Exeter, Exeter, United Kingdom

**Keywords:** Bayesian integration, grasping, grip force, load force, weight perception

## Abstract

The material-weight illusion (MWI) occurs when an object that looks heavy (e.g., stone) and one that looks light (e.g., Styrofoam) have the same mass. When such stimuli are lifted, the heavier-looking object feels lighter than the lighter-looking object, presumably because well-learned priors about the density of different materials are violated. We examined whether a similar illusion occurs when a certain weight distribution is expected (such as the metal end of a hammer being heavier), but weight is uniformly distributed. In *experiment 1*, participants lifted bipartite objects that appeared to be made of two materials (combinations of stone, Styrofoam, and wood) but were manipulated to have a uniform weight distribution. Most participants experienced an inverted MWI (i.e., the heavier-looking side felt heavier), suggesting an integration of incoming sensory information with density priors. However, a replication of the classic MWI was found when the objects appeared to be uniformly made of just one of the materials (*experiment 2*). Both illusions seemed to be independent of the forces used when the objects were lifted. When lifting bipartite objects but asked to judge the weight of the whole object, participants experienced no illusion (*experiment 3*). In *experiment 4*, we investigated weight perception in objects with a nonuniform weight distribution and again found evidence for an integration of prior and sensory information. Taken together, our seemingly contradictory results challenge most theories about the MWI. However, Bayesian integration of competing density priors with the likelihood of incoming sensory information may explain the opposing illusions.

**NEW & NOTEWORTHY** We report a novel weight illusion that contradicts all current explanations of the material-weight illusion: When lifting an object composed of two materials, the heavier-looking side feels heavier, even when the true weight distribution is uniform. The opposite (classic) illusion is found when the same materials are lifted in two separate objects. Identifying the common mechanism underlying both illusions will have implications for perception more generally. A potential candidate is Bayesian inference with competing priors.

## INTRODUCTION

A lifetime of experience has taught us about the typical properties of objects and materials. Thus, by only looking at a brick, we expect it to be heavy, although weight is not per se a visual property. This enables us to adjust our behavior in an anticipatory fashion (Westling and Johansson 1984); we use more force to lift a stone brick than one made of Styrofoam and choose appropriate points on the objects to grasp them (Paulun et al. 2016). The material-weight illusion (MWI) is a striking example of how visually evoked expectations about material properties can influence heaviness perception in a top-down manner. The MWI can be experienced when lifting objects of equal size and shape that visually appear to be made of materials that substantially differ in density, such as brass and Styrofoam (but which have been manipulated to have the same mass). Although their mass is physically identical, these objects feel as though they differ in weight when lifted one after the other; the heavier-looking object feels lighter, whereas the lighter-looking object feels heavier. This illusion has been known at least since the late 19th century (Seashore 1899; Wolfe 1898), and it has been replicated multiple times in various versions (Baugh et al. 2012; Buckingham et al. 2009, 2011; Buckingham and Goodale 2013; Ellis and Lederman 1999; Vicovaro and Burigana 2017).

A key component of the illusion is strong prior expectations about the density of different materials, e.g., stone, metal, wood, or Styrofoam. If a material is known only to a specific population, a weight illusion will be experienced only by that group of participants (golf-ball illusion; Ellis and Lederman 1998). Weight expectations that lead to an MWI can be evoked through touch alone (Ellis and Lederman 1999), vision alone (Buckingham et al. 2011), or a combination of both (Ellis and Lederman 1999). These expectations are related to (implicit) long-term priors and are not altered during an experiment. Thus, the MWI occurs not only when an object is lifted for the first time but repeatedly over the course of many trials (Buckingham et al. 2009). In other words, even after lifting a “heavy” Styrofoam object several times, participants neither adjust their expectations nor their long-term prior; it continues to feel even heavier than an equally weighted stone object. This leads to another key component of the MWI, the violation of weight expectations; the weight force of a material is larger or smaller than expected. Interestingly, this violation of expectations leads to a perceptual contrast effect. A heavy piece of Styrofoam is perceived as not only unexpectedly heavy but even heavier than an equally weighted object of a different material. This is in stark contrast to a large body of research on cases in which prior knowledge and sensory information are integrated by the perceptual system (e.g., see Adams et al. 2004; Ernst and Bülthoff 2004; Kersten and Yuille 2003; Körding et al. 2004; Körding and Wolpert 2004; Langer and Bülthoff 2001; Sun and Perona 1998; Weiss et al. 2002). Bayesian integration would predict that contradicting prior and sensory information (e.g., a heavy object with a Styrofoam surface) would be integrated to a perceived weight that lies somewhere between the two. Even “robust estimation,” when the cue conflict is large (Landy et al. 1995), would predict that observers would rely solely on the more reliable modality (i.e., either the felt weight or the visually expected weight) rather than a contrast effect in which the perceived weight is outside the range between the prior and the sensory information. As a result, weight illusions like the MWI or the related size weight illusion (SWI) have been termed “anti-Bayesian” (Brayanov and Smith 2010). What is the advantage of such anti-Bayesian behavior? Baugh et al (2012) speculated that if an object strongly contradicts the prior expectation about a material class, this object is not incorporated into the prior but marked as an outlier by the perceptual system (hence, it is contrasted and feels even lighter/heavier). Incorporating outliers into the prior, by contrast, would make the prior more unreliable. Only long-term exposure to unexpectedly weighted objects/materials, when they become the rule and not the exception, may lead to an adjustment of the long-term prior (and can even invert a weight illusion, as has been shown for the SWI; see Flanagan et al. 2008). The anti-Bayesian view on weight illusions has been challenged by Peters et al. (2016), who argued that the SWI can indeed be explained by Bayesian integration if one incorporates the possibility of multiple competing density priors, and by Wolf et al. (2018), who argued that the SWI can be explained by maximum-likelihood integration of mass and density estimates with correlated noise.

In contrast to the unchanging perceptual illusion, the motor system adjusts grip and load forces quickly to the actual mass of the objects within few trials (Buckingham et al. 2009). This dissociation between perception and action shows that the MWI cannot purely be the result of a sensorimotor mismatch between the applied force (scaled according to the expected weight) and the true physical weight. It has been suggested that long-term priors and short-term sensorimotor memories interact when equally weighted objects made of different materials are lifted, resulting in the MWI (Baugh et al. 2012).

Unlike some experimental settings, our world is not filled with homogeneous objects made from pure metal, wood, or Styrofoam; rather, objects are often composed of multiple materials, such as hammers, scissors, and lollipops. In this case, the mass will not be distributed equally within the object. If all of the materials comprising such an object are familiar, we can presumably infer the likely weight distribution. For example, we would expect the metal end of a hammer to be much heavier than the wooden end and thus for its center of mass (CoM) to be closer to the head. Indeed, Crajé et al. (2013) showed that humans can accurately judge the CoM location from visual density cues in asymmetric objects. However, knowledge of the CoM location in objects with nonuniform density did not enable participants of that study to anticipatorily scale the initial fingertip forces to prevent object tilt. Instead, participants required lifting the object several times to learn how to prevent an initial tilt. Thus, there seems to be a dissociation of how a mass distribution is represented in the perceptual and motor system. Do violations of an expected weight distribution also lead to an illusion, much as unexpected weights result in the MWI? For the MWI, the relevant sensorimotor information originates from the mass of the object and the force required to lift that mass. In contrast, differences in mass distribution would be signaled through other types of information, such as a torque (the rotational equivalent of force), which rotates the object toward its heavier side. Weight perception not only depends on the mass of the object but also varies depending on the first moment of mass (Kingma et al. 2002). Here, we ask whether sensorimotor information, such as torque, leads to weight illusions localized to specific parts of the object. We systematically investigated these questions by violating the expected mass distribution in bipartite-looking objects (composed of 2 materials) and asking participants to report their apparent weight and CoM before and after lifting them. In *experiment 1*, the mass distribution was manipulated to be uniform in objects for which participants expected a nonuniform mass distribution. This led to an unexpected inversion of the MWI. *Experiment 2* was conducted to confirm that this effect was due to the violations of expected mass distribution and corresponding sensory information (torque, more specifically its absence) and not to other features of the objects used in *experiment 1*. *Experiment 3* tested whether judging the overall weight (instead of the weight distribution) of bipartite objects would elicit an inverted or classic MWI. We found that in this case participants do not experience any weight illusion. Finally, in *experiment 4*, we used objects with a nonuniform mass distribution to test whether the effects observed in *experiment 1* were related to the lack of any torque signal. More specifically, we tested weight perception in objects that appeared to be uniform visually but were manipulated to have a nonuniform mass distribution as well as in objects that were expected to have nonuniform mass in a way that was discrepant from the visual appearance. Thus, unlike *experiment 1*, there was actually a torque signal present in *experiment 4*.

## MATERIALS AND METHODS

### Experiment 1

#### Participants.

Fifty-three students (39 females, 14 males) from the University of Western Ontario took part in *experiment 1*. All were right-handed by self-report, and the average age was 21 yr (SD = 4 yr). All participants were naive with regard to the aims of the study and gave written, informed consent before the experiment. The procedure was approved by the ethics board at the University of Western Ontario and in agreement with the declaration of Helsinki. Students were compensated with 10 CAD for their participation. Two participants were excluded from the analysis because of missing data, and two other participants were excluded because they did not understand the instructions and hence, were unable to complete the task properly. More specifically, one participant did not understand what the CoM of an object was, which was a prerequisite for performing the task, and one participant did not always use the right hand as instructed. Thus, the data of 49 participants were used for data analysis.

#### Stimuli.

**Fig. 1.**
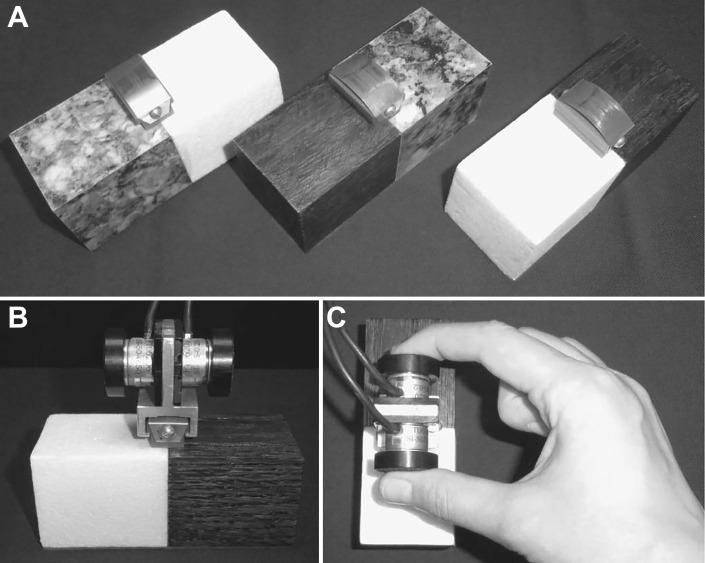
Stimuli used in *experiment 1*. *A*: the 3 bipartite objects, with halves that appeared to be made of different materials: granite, Styrofoam, and wood. *B*: two 6-axis force-torque transducers were attached centrally to the objects on a small handle. *C*: an object grasped with a precision grip as in the experiment.

Three bipartite objects served as stimuli in our first study (see Fig. 1*A*). All had the same size (4 × 4 × 10 cm) and looked as if their two halves were made of different materials: stone and wood, wood and Styrofoam, or Styrofoam and stone. The objects were carved out and partially filled with lead and their bases coated with fleece to reduce auditory cues when the objects were placed. Thus, they all had the same mass (400 *g*), which was distributed evenly around the geometric center of the objects. A small handle was attached centrally on top of the objects, onto which the force transducers could be mounted and removed on every trial. A pair of six-axis force-torque (F/T) sensors (Nano17 F/T; ATI Industrial Automation, Garner, NC) were built into a small handle with opposing grip pads (see Fig. 1*B*). These grip pads had a diameter of 2.5 cm and were covered with black sandpaper and thus allowed a comfortable precision grip of index finger and thumb. The handle with the transducer added another 50 g to the weight of the objects. The configuration of the grip pads and thus the force transducers was such that the index finger would be on one half, i.e., one material, of the object and the thumb would be on the other side, i.e., the other material (see Fig. 1*C*). For the practice trials, we used an object with the same dimensions, weight, and mass distribution as the bipartite objects, but with uniform dark wood appearance.

#### Set up and procedure.

Participants were seated in front of a small table that was covered with black cloth. All objects that were used in the task were placed on the table before the experiment. At each trial, participants were instructed to place their right (dominant) hand on the table and close their eyes while the experimenter placed one of the objects in front of them. The objects were placed with one of the short sides facing the participants; i.e., one material was closer to them than the other one. The orientation of each object was kept constant within participants and counterbalanced between individuals. However, a given participant did not always face the heavier (or lighter) looking material for all three objects. At each trial, a computer-generated “beep” signaled to the participants to open their eyes and start the movement. Their task was to grasp the object at the grip pads with a precision grip of index finger and thumb, lift the object to a comfortable height (∼15–20 cm above the table), and hold it stable without hefting it or letting it rotate or fall. After 3 s, another beep occurred, which was the signal to place the object back onto the table. Forces and torques were measured during the 3 s between the two signals at 1,000 Hz. The movement was performed at a self-chosen, natural speed. A perceptual measure of the weight of both halves of the object was taken after each lift. Importantly, a perceptual judgment of the weight of the objects’ halves was also acquired before each object was lifted for the first time, i.e., based solely on the visual appearance of the objects to gain insight into participants’ prior expectations.

The type of perceptual judgment varied between participants. Twenty-four participants were asked to give a numerical rating of how heavy each half of the object felt after each lift, in addition to how heavy they thought it would feel before the experimental lifting trials. We counterbalanced across participants which half of each object they rated first. Participants were asked to give their rating on an arbitrary scale, with the only constraint that larger numbers should represent heavier weights (absolute magnitude estimation; see Zwislocki and Goodman 1980). The other 25 participants were asked to indicate the perceived CoM of the objects as a more implicit measure of the perceived mass distribution. It has been shown that observers can accurately judge the CoM of two- and three-dimensional objects using symmetry (Bingham and Muchisky 1993a, 1993b) or density cues (Crajé et al. 2013). If they perceived both halves of the object to be equal in weight, they should report the CoM to be at the geometric center of the object. If they perceived one or the other side to be heavier, this would result in a shift of the perceived CoM toward that side. To obtain the perceived CoM, participants pointed with the sharpened end of a wooden stick (like a pencil) to the perceived CoM along the elongated side of the object, similar to the task by Crajé et al. (2013). The experimenter recorded this measure by using a small ruler that was placed next to the object as soon as the participant had made his/her judgment. Every participant completed five practice trials with the uniform wooden block (more if necessary), followed by 30 trials with the bipartite objects. Objects were presented in one of six different pseudorandom orders so that each object was lifted 10 times, and all three objects were lifted before any were repeated.

#### Data analysis.

The numerical heaviness ratings were transformed into *z*-scores based on the mean and SD of each individual participant (practice and main trials). The CoM judgments provided one number instead of a separate rating for each material. Thus, we used the judged CoM (in cm) as a rating for one material and subtracted the judged CoM from 10 cm (the length of the object) to gain a rating for the other half of the object. This was done so that the larger number resulted for the material at the side where the CoM was perceived, i.e., as in the other group of participants, the larger the number, the heavier that material was perceived. The resulting CoM judgments are inherently on the same scale (between 0 and 10) for all participants, but to compare these judgments to the ratings of the other group we also transformed these values into *z*-scores (based on the mean and SD of each individual). These *z*-scores were used in our statistical analysis. The core question of this experiment was whether there were differences in the expected as well as perceived weight of the differently looking halves of the objects. Therefore, we averaged the ratings of the perceived weight for each participant and material to calculate a material (stone vs. wood vs. Styrofoam) × lift (before vs. after) × task (numerical rating vs. CoM judgment) mixed-design ANOVA across all participants. We corrected for violations of sphericity where necessary and report the Greenhouse-Geisser corrected values. Pairwise post hoc comparisons were Bonferroni corrected.

To determine the strength of the illusion on an individual basis, for each participant we calculated the average rating for Styrofoam and stone after lifting and subtracted the resulting Styrofoam value from the stone value (Idx_MWI_ = Ψ_Stone_ − Ψ_Styrofoam_). The same was done for the individual ratings before lifting, i.e., their priors. Positive values of this index indicate that stone is perceived/expected heavier than Styrofoam, whereas negative values indicate that stone was perceived/expected lighter than Styrofoam. A two-sided *t*-test was performed to test whether the illusion index was significantly different from zero after lifting.

**Fig. 2.**
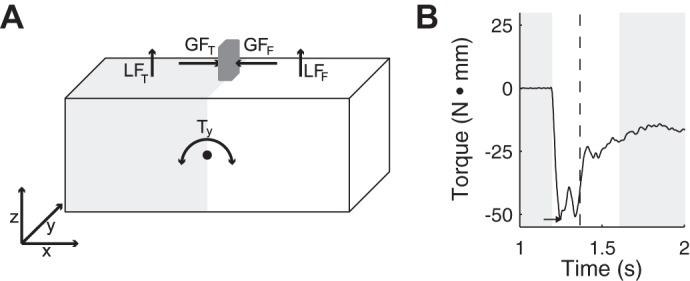
*A*: sketch of a bipartite object in the 3-dimensional (3D) coordinate system. Load force (LF) was calculated for each sensor (i.e., finger) as force in the z-direction, and grip force (GF) as force in the *x*-direction. Torque was calculated as rotational force around a pivot point at the center of mass (CoM) of the object. *B*: filtered torque data around the *y*-axis from one example trial (thumb side had stone appearance, finger side had wood appearance). White area indicates the loading phase. We used the first local extremum as dependent variable, indicated by the small arrow. This object was initially rotated toward the lighter looking side. The vertical dashed line shows the moment of liftoff (when LF > weight force of the object). GF_F_, grip force of the finger; GF_T_, grip force of the thumb; LF_F_, load force of the finger; LF_T_, load force of the thumb.

Data of the F/T transducers were first transformed into one common coordinate system (see Fig. 2*A*) such that the long side of the object corresponded to the *x*-dimension (i.e., *x* is normal to the grip surfaces), the short side of the object corresponded to the *y*-dimension, and *z* was orthogonal to the *x-y* plane. Furthermore, data from one group of participants were rotated and relabeled so that the force data could be analyzed irrespective of the orientation of the objects (which we had counterbalanced between participants).

When an object with one heavy and one light side is lifted, there are at least four strategies to prevent the object from tilting: *1*) increasing the grip force (GF) at the heavy side, *2*) increasing the load force (LF) at the heavy side, *3*) keeping forces the same but applying the center of pressure at different heights (higher on heaver side), or *4*) any combination of these. All strategies can counteract a torque emerging from a nonuniform weight distribution or, in turn, can cause a torque if there is no weight difference between the two halves (as in our experiment). If participants employ such strategies in an anticipatory fashion, we expect to find an initial torque when the objects are lifted.

Torque (τ) is the cross-product between a force vector (F) and a distance vector connecting the CoM and the point of force application (r). We calculated the cross-product between the applied force of the thumb and the distance between its center of pressure (CoP) and the CoM (τ_thumb_ = F_thumb_ × r_thumb_) and likewise for the index finger (τ_index_ = F_index_ × r_index_). The vertical CoP of each digit was calculated following Zhang and colleagues (2010) and adapted to the orientation of the sensors in our setup. Furthermore, we calculated the cross-product between the weight force of each object’s half and its distance to the CoM (τ_half1_ = F_half1_ × r_half1_ and τ_half2_ = F_half2_ ×r_half2_). The overall torque is simply the sum of these four cross-products (τ = τ_thumb_ + τ_index_ + τ_half1_ + τ_half2_). Central to our investigation was the torque around the *y*-axis (see Fig. 2*A*). Again, we would expect a torque only around *y* in the initial stage of the movement, because there was no actual weight difference within the objects (τ_half1_ + τ_half2_ = 0 in *experiment 1*), and a resulting overall torque should thus be corrected. Therefore, we analyzed torque only during the loading phase of the movement. The beginning of the loading phase was determined by combining multiple criteria [similar to the MSI method proposed by Schot and colleagues (2010)]. We selected the first time point at which the GF of at least one finger and the LF of at least one finger were above a threshold (0.01 N), and the absolute torque around the *y*-axis exceeded 1.5 N·mm. The GF of each digit was the force measured in the *x*-dimension, with the finger’s GF multiplied by −1 (because the 2 digits act in opposite directions; see Fig. 2*A*). The LF was defined as the force in the *z-*direction (see Fig. 2*A*). The end of the loading phase was defined as the first point in time after the initial peak in which the total LF (sum of both digits) fell below the weight force of the object or (if not reached) below the median LF.

The torque signal was smoothed with a fourth-order, zero-phase lag, low-pass Butterworth filter with a cutoff frequency of 50 Hz. We used the first local extremum during the loading phase as our dependent variable (see Fig. 2*B*). Its sign tells in which direction the object was rotated initially (i.e., toward the heavier- or lighter-looking material), and its value indicates the amount. To simplify interpretation, we aligned the torques across different orientations of each object such that positive torques always corresponded to rotations toward the heavier-looking side and negative torques toward the lighter-looking side. If participants expected one half to be heavier and modified their grip in an anticipatory fashion, we would expect an initial torque in the direction of the lighter-looking side.

We calculated an object (stone-wood vs. Styrofoam-stone vs. Styrofoam-wood) × lift (first vs. subsequent lifts) − repeated-measures ANOVA for the peak torque. We corrected for violations of sphericity where necessary and report the Greenhouse-Geisser corrected values. Data from all experiments can be downloaded here: https://doi.org/10.5281/zenodo.1345746.

### Experiment 2

In *experiment 1*, we found a new and unexpected inversion of the MWI. Is this illusion down to something unique about how we deal with bipartite objects? Or, rather, is it due to some trivial properties of our stimuli, e.g., their specific shape, or the lifting task? *Experiment 2* was conducted to test whether we could replicate the classic MWI (e.g., Buckingham et al. 2009) using the same materials, weights, and shapes as in our first experiment but in uniform objects. More specifically, we wanted to exclude the possibility that any of the objects’ properties, except for the fact that they are bipartite, could explain our results of the first experiment.

#### Participants.

Twenty-four students (6 men, 18 women) of the University of Western Ontario participated in *experiment 2*, none of whom had participated in *experiment 1*. They were on average 20 yr old (SD = 3 yr) and right-handed by self-report. All were naive to the aims of the study and gave written, informed consent before their participation. Students received 10 CAD for taking part in the experiment. The experimental procedure was approved by the ethics board at the University of Western Ontario and in agreement with the Declaration of Helsinki.

#### Stimuli.

Three objects served as stimuli in *experiment 2* (see Fig. 5*A*). They had the same shape, size, and weight as the ones in *experiment 1*, but here they appeared to be made from only one of our materials (Styrofoam, wood, granite-like). The same central handle containing the force/torque transducers as in *experiment 1* was attached to these objects.

#### Setup and procedure.

Set up and procedure were mostly the same as in *experiment 1.* The main difference was that participants did not have to rate the heaviness of the individual halves of the objects but each object as a whole. Thus, no group of participants performed a CoM judgment; all gave numerical ratings of heaviness. In short, participants were instructed, and then they rated the weight of each object based on visual information alone and completed five practice trials with the wooden object and, finally, 10 pseudorandomly interleaved trials with each object, i.e., 30 trials.

#### Data analysis.

As in *experiment 1*, perceptual ratings were transformed into *z*-scores, and post-lifting scores were averaged for each participant and material. Data were then analyzed with a material (stone vs. wood vs. Styrofoam) × lift (before vs. after) − repeated-measures ANOVA. Additionally, we calculated an illusion index for each participant as in *experiment 1* and used a one-sample *t*-test to test whether it was significantly different from zero after lifting.

Preprocessing of the data from the F/T transducers was done exactly as in *experiment 1*. Instead of torque, we were interested in the effects on GF and LF and their rates of change. The GF of each digit was the force measured in the *x*-dimension, with the finger’s GF multiplied by −1 (because the 2 digits act in opposite directions). We used the mean of both GF signals. We determined the first peak of GF as well as its peak rate of change (GFR) as dependent variables. To determine the first peak, we used the derivative of the smoothed force signal (smoothed with a Gaussian filter, σ = 30 ms) to identify the first local extrema. More specifically, we determined the point in time at which 70% of the maximum of the derivative was reached and the first point in time at which the signal became negative after this (or the end of the trial, if it never became negative). In the period between these two time points, we determined the first local maximum and minimum. We then determined the maximum of the original force signal in the time between the first local maximum and minimum; this was the peak GF used in further analysis. We determined the GF rate of change by smoothing the force signal with a fourth-order, zero-phase lag, low-pass Butterworth filter with a cutoff frequency of 50 Hz and then differentiating the signal. We calculated the peak of this function as a dependent variable, i.e., the maximal slope of the original force signal.

The LF was defined as the force in the *z-*direction. We used the mean of LF of both fingers and determined the first peak and its peak rate of change (LFR) with the same method as for the GF. We calculated a material (stone vs. wood vs. Styrofoam) × lift (first vs. subsequent lifts) repeated-measures ANOVA for these four measures. We corrected for violations of sphericity where necessary and report the Greenhouse-Geisser corrected values. Pairwise post hoc comparisons were Bonferroni corrected.

### Experiment 3

In *experiment 1*, we found an inverted MWI when participants judged the masses of each half of bipartite objects. In *experiment 2* we found the classic MWI when participants judged the entire mass of uniform objects. In *experiment 3*, we asked participants to lift bipartite objects (as in *experiment 1*) and estimate the weight of the entire object (as in *experiment 2*). With this manipulation, we aimed to test whether bipartite objects would invert the MWI when participants were not explicitly required to make judgments of the mass distribution but of the overall mass instead.

#### Participants.

Twenty-four students (5 men, 19 women) of the University of Giessen participated in *experiment 3*. They were on average 22 yr old (SD = 3 yr). Three participants were left-handed by self-report, and all participants used their dominant hand for the task. All participants were naive to the aims of the study. They gave written, informed consent before the experiment and received 8€/h for their participation. *Experiment 3* was approved by the local ethics committee and was in agreement with the Declaration of Helsinki.

#### Stimuli.

The same objects that were used in *experiment 1* served as stimuli in *experiment 3*.

#### Set up and procedure.

Set up and procedure were almost exactly as in *experiment 1* (numerical heaviness rating group) with two differences: 1) Participants were never asked to rate the weight of the halves of the objects. Instead, they were asked to rate the apparent weight of each object as a whole. 2) In this Experiment we did not collect force and torque data, but instead used a sham version of the handle that did not contain the F/T transducers. This was done because we had not found any effect on the F/T data in *experiment 1* when participants were lifting the exact same objects.

#### Data analysis.

Data analysis was done in the same ways as in *experiments 1* and *2*. Perceptual ratings of each participant were transformed into *z*-scores, and post-lifting scores were averaged for each participant and object. Data were then analyzed with an object (stone-wood vs. Styrofoam-stone vs. Styrofoam-wood) × lift (before vs. after) − repeated-measures ANOVA. We corrected for violations of sphericity where necessary and report the Greenhouse-Geisser corrected values. Pairwise post hoc comparisons were Bonferroni corrected. To determine the strength of the illusion on an individual basis, for each participant we calculated the illusion index similar to the previous experiments. Instead of calculating it by subtracting ratings for the lightest-looking from the heaviest-looking material (stone – Styrofoam), here we subtracted the ratings of the lightest-looking object from the heaviest-looking object (stone-wood – Styrofoam-wood). Thus, interpretation of the resulting indices is in line with the illusion index in the previous experiments. A two-sided *t*-test was performed to test whether the illusion index (after lifting) was significantly different from zero. Two independent *t*-tests were performed to test whether the illusion index in *experiment 3* was different from the illusion index in *experiments 1* and *2*. α-Levels were adjusted for multiple comparisons.

### Experiment 4

*Experiment 1* demonstrated an inverted MWI illusion for bipartite objects when there was no real difference in weight between the two halves. In *experiment 4*, we sought to measure how this illusion interacted with real differences in mass in both the expected and unexpected direction. More specifically, *experiment 4* complements *experiment 1* in two ways. First, in *experiment 1* the objects had a uniform mass distribution but were expected to have a nonuniform distribution, whereas in *experiment 4* the opposite was the case. Objects had a nonuniform mass distribution but were expected to have either a uniform distribution or a nonuniformity in a different direction. Second, *experiment 1* was characterized by the absence of an expected torque signal, whereas in *experiment 4* there is a torque signal present (in most cases). This allows us to test whether the inversion of the classic MWI observed in *experiment 1* is due to the lack of torque-related sensory signals.

#### Participants.

Twenty-four students (15 men, 9 women) of the University of Western Ontario participated in *experiment 4*. They were on average 25 yr old (SD = 7 yr). All were right-handed by self-report and naive to the aims of the study. Students gave written informed consent before the experiment and received 10 CAD afterwards for their participation. *Experiment 4* was approved by the ethics board at the University of Western Ontario and was in agreement with the Declaration of Helsinki.

#### Stimuli.

Five objects served as stimuli in *experiment 4*, four of which included a weight difference of 100 g between the two halves. We chose a weight difference of 100 g because this is similar to the difference that participants perceived on average in *experiment 1*. For a 400-g object, a CoM shifted 0.82 mm to one side (as we found for the Styrofoam-stone object in *experiment 1*) transfers to a weight difference of 128 g between the two halves. Therefore, we wanted to test how participants would perceive a weight difference of 100 g within one object.

Three of the objects were bipartite; they appeared to be made of stone and Styrofoam. In one of these, the Styrofoam-side was artificially made 100 g heavier than the stone side (250 vs. 150 g); i.e., the weight distribution was in the unexpected direction. In another bipartite object, the weight distribution was in the expected direction (although the difference was not as large as it would be for real materials); i.e., the stone side was 100 g heavier than the Styrofoam side. To be able to make within-participant comparisons, we additionally used the stone-Styrofoam object from *experiment 1*, i.e., with an equal weight distribution. We also had one object that appeared to be uniformly made of stone but contained a weight difference of 100 g between the two halves, as well as one object that appeared to be uniformly made of Styrofoam, but contained the same weight difference. We chose only to use stone and Styrofoam in *experiment 4* to reduce the number of objects and because they produced the largest effects in *experiments 1* and *2*.

#### Set up and procedure.

Set up and procedure were the same as in *experiment 1*, and participants had to give numerical heaviness ratings of the halves of the objects. Different from *experiment 1*, not all objects were placed on the table before the experiment, only the object that was judged during a given trial. Before the experiment, participants rated the expected heaviness of the halves of the two uniform-looking objects and of one bipartite object. Because the three bipartite objects were visually identical, we did not obtain separate ratings of the prior expectations for them. We counterbalanced between participants which bipartite object was rated before lifting. In short, participants were instructed, and then they rated the weight of the halves of two uniform objects and one bipartite object based on visual information alone and then completed five practice trials with the wooden object and, finally, 10 pseudorandomly interleaved trials with each object, i.e., 50 trials.

#### Data analysis.

As in *experiments 1* and *2*, perceptual ratings were transformed into *z*-scores. In this experiment, post-lifting scores were averaged for each participant, object half, and object. To determine whether participants expected and perceived a weight difference in each of the objects, we calculated a paired-sample *t*-test for each object to compare the ratings of both halves. We compared the strength of significant effects in different objects by determining the average difference score (between object halves) for each participant and calculating paired-sample *t*-tests. Bonferroni correction was applied in case of multiple comparisons.

We used the same setup with the F/T transducers in this experiment as in the first two to keep everything as comparable as possible. Here, however, we were interested mostly in the perceptual effects. Unlike the other two experiments, in which the motor system could in principle learn the weight (distribution) over the course of the experiment due to a fixed association between a given material and its weight, the material was not diagnostic for the weight in *experiment 4* because identical-looking halves varied in weight. Therefore, we did not predict any specific effect on the initial force measures. However, we were interested in how participants would counteract real weight differences when lifting the objects. Therefore, we investigated the initial torque during the loading phase as well as the median of the torque signal during the holding phase. Preprocessing of the F/T data was carried out in the same way as in *experiment 1*. To simplify interpretation, we aligned the torques across different orientations of each object such that positive torques always corresponded to rotations toward the heavier side and negative torques toward the lighter side. In case of the bipartite object with a uniform weight distribution, i.e., where no side was heavy, we aligned the torques measures so that a torque toward the heavier-looking side is positive. For statistical analysis, we used one-sample *t*-tests to test whether the mean was different from zero for the torque measures. α-Levels were adjusted for multiple comparisons, following the Bonferroni method.

## RESULTS

### Experiment 1

#### Perception.

**Fig. 3.**
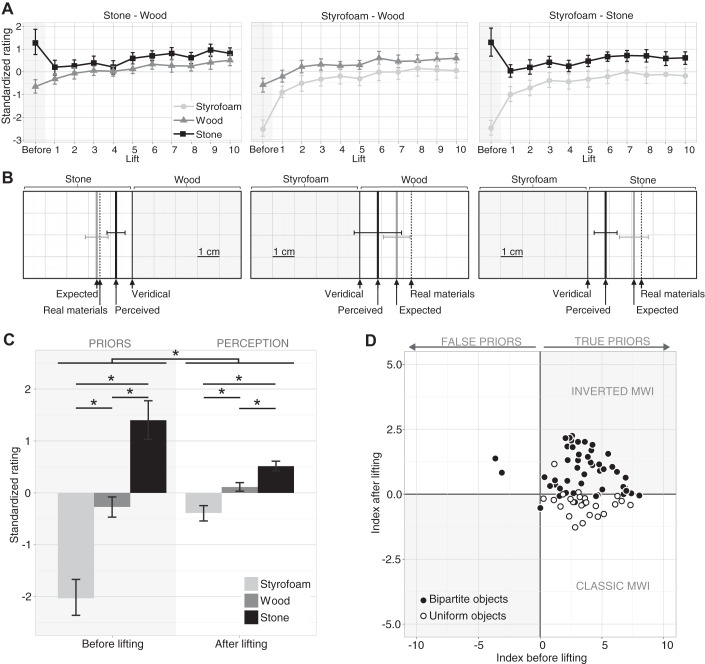
Perceptual results of *experiment 1*. *A*: mean standardized heaviness ratings for each material (color) before lifting (shaded area) and after each subsequent lift in separate plots for each object. Data are averaged across participants, who gave a numerical heaviness rating; error bars show 95% confidence intervals. *B*: side views of the 3 objects together with the horizontal position of the veridical center of mass (CoM; thin black line), the position at which the CoM would be if the materials were real (dotted line), and the mean expected CoM position (as rated before lifting; gray line) and perceived CoM position (after lifting; thick black line). Data were averaged across trials and participants, who were asked to judge the CoM. Error bars show 95% confidence interval between participants. *C*: standardized ratings averaged for each material across all participants, trials, and objects before (shaded area) and after lifting. *Significant differences between the perceived heaviness of the materials as well as between the perceived heaviness before and after lifting. *D*: illusion index before vs. after lifting (perceived heaviness of stone − Styrofoam) for each participant in *experiment 1* (●) and *experiment 2* (○), in which uniform-looking objects with a stone or Styrofoam appearance were used as stimuli to induce the classic material-weight illusion (MWI). Please note that the *x*- and *y*-axes are scaled differently here. This was necessary because the perceived differences (*y*-axis) are smaller than the expected differences (*x*-axis). Participants in the *top right* (and *bottom left*) quadrant experienced the inverted MWI (gray fields), whereas participants who experienced the classic MWI (white fields) fall in the *bottom right* (and *top left*) quadrant.

Figure 3*A* shows the averaged standardized numerical ratings for the different materials and objects, respectively. Unsurprisingly, and irrespective of the object, stone was expected to be heavier than wood and wood heavier than Styrofoam. Interestingly, and in contrast to the standard MWI, even after participants had lifted the objects, they on average continued to experience stone as feeling heavier than wood and wood as feeling heavier than Styrofoam. In fact, all materials had the same weight, so any perceived differences were illusory. This illusory weight difference was smaller than the difference in participants’ pre-lift expectations but remained present over the course of the experiment.

A similar pattern of results was observed for the group of participants judging the perceived horizontal CoM location. Figure 3*B* shows a sketch of the side view of each object. The veridical CoM was always at the geometric center of the object. The dotted lines show the locations where the CoM would lie if the materials were real granite, oak wood, and Styrofoam. Interestingly, participants (on average) expected the CoM of each object (gray thick line) to be very close to the CoM of real materials, suggesting that they have good internalized representations of the relative densities of materials. After the objects were lifted, the perceived CoM shifted toward the veridical CoM but still remained on the side of the heavier-looking material (i.e., the heavier-looking material was reported to be heavier).

Fig. 3*C* shows the average expected and perceived weight of the three materials from all participants. The material ×lift × task mixed-design ANOVA confirmed the above observations with a significant main effect of material (statistics can be found in Table 1). Styrofoam was rated significantly lighter [−1.21 ± 0.09 (means ± SE)] than stone (0.96 ± 0.10) and wood (−0.04 ± 0.07) and wood significantly lighter than stone (all *P* < 0.001, adjusted α = 0.0167). Ratings before lifting were significantly lower (−0.31 ± 0.06) than after lifting (0.11 ± 0.03). Although all materials had the same weight, they were not only expected but also perceived to differ in their weight. That means our objects induced a weight illusion but in the opposite direction of the classic MWI. The ANOVA also revealed a significant interaction such that the difference between the materials was larger before than after lifting, i.e., the weight difference was expected to be larger than it felt.

**Table 1.** Results of the mixed-design and repeated-measures ANOVAs of experiment 1

Measure (Factor)	*df_1_*	*df_2_*	*F*	*P* Value
Heaviness rating				
Material	1.52	71.23	122.10	<0.001*
Lift	1	47	26.01	<0.001*
Task	1	47	17.18	<0.001*
Material × Lift	1.33	62.41	38.34	<0.001*
Material × Task	1.52	71.2	0.42	0.656
Lift × Task	1	47	19.07	<0.001*
3-way interaction	1.33	62.41	1.28	0.283
Heaviness rating				
Object	2	46	77.24	<0.001*
Lift	1	23	24.98	<0.001*
Object × Lift	1.31	30.09	45.42	<0.001*
Peak torque Y				
Object	2	96	0.82	0.442
Lift	1	48	0.69	0.410
Object × Lift	1.51	72.24	0.22	0.736

* Significant effects in the ANOVAs.

Because we used two different perceptual measures, we were interested in whether we would find a difference between the two groups and introduced this as a third factor in our ANOVA. Indeed, we found a main effect of judgment type. Numerical ratings resulted in on average smaller values (−0.22 ± 0.44) than the CoM judgments (0.03 ± 0.04). Furthermore, we found a significant interaction between task and lift: The difference between expectation and perception was larger for the group that gave a numerical rating. There was no interaction between material and task and no three-way interaction between all factors. Whether the differences between the two tasks are related to perceptual differences, the different response format, or the different judgment type (e.g., judging a ratio or 2 independent judgments) or simply due to the fact that the response range was limited in one (CoM judgment) but not the other task is not clear from our data.

To determine the strength of the illusion on an individual basis, we calculated an illusion index for each participant. Figure 3*D* shows this index before and after lifting for each participant. The overwhelming majority of our 49 participants both expected and perceived stone to be heavier than Styrofoam; i.e., they experienced an inverted material-weight illusion (Fig. 3*D*, *top right*). Some participants experienced no illusion after lifting (points that lie on the horizontal axis), and only one participant had a negative illusion index after lifting. A two-sample *t*-test showed that, overall, the illusion index after lifting was significantly larger than zero [*t*(48) = 8.03, *P* < 0.001].

In summary, our results show that bipartite objects that appear to be made of different materials, but which in reality have a uniform mass distribution, elicit a strong weight illusion. In contrast to the well-known MWI for uniform objects, bipartite objects lead to an inverted illusion in which heavier-looking materials feel heavier and lighter-looking materials feel lighter. Thus, prior expectations and sensory information about weight seem to have been integrated into a common heaviness percept.

#### Torque.

**Fig. 4.**
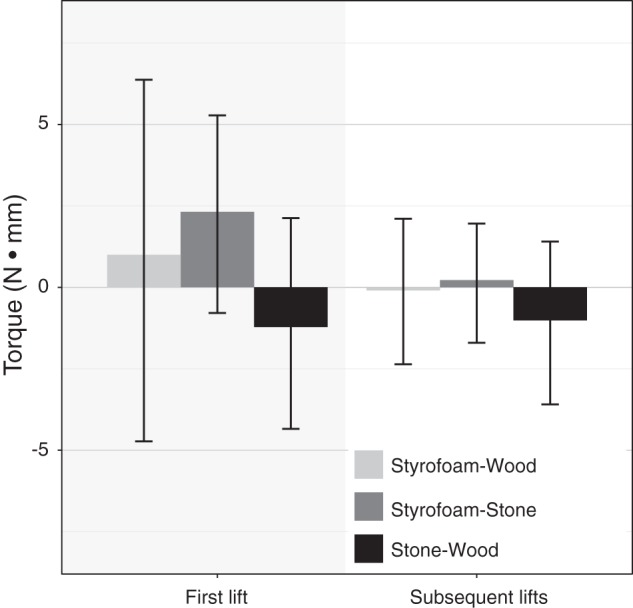
Mean peak torque around the *y*-axis in first (shaded) and subsequent lifts. An initial rotation toward the lighter-looking side is indicated by negative values; positive values indicate a rotation toward the heavier-looking side. No rotation would result in a torque of zero. Error bars show 95% confidence intervals.

Previous studies on the material (Buckingham et al. 2009) and size weight illusion (Flanagan and Beltzner 2000) found differences in load or grip force measures based on objects’ visual appearance only in the first trial (not subsequent trials), because the motor system must rely on prior expectations based on the visual appearance of the object in the first but not in later trials. Thus, we were expecting a similar pattern for the measured torque. More specifically, we would expect a negative torque in the first trial and no torque in later trials. However, we did not find an effect of object, lift, or their interaction on torque (see Fig. 4). An additional one-sample *t*-test showed that the net torque was not significantly different from zero [*t*(48) = −0.35, *P* = 0.731].

Thus, contrary to the perceptual illusion, there was no effect of the visual appearance of the objects on the motor system. There are several possibilities for the discrepancy between perceived weight and weight expectations as measured through applied forces and resulting torque. The two systems could rely on different types of information, whereby the motor system seems to have access to more accurate information in this case. Another possibility is that materials are not an effective cue for producing an anticipatory torque. Salimi et al. (2003) investigated how well lifting forces could be adjusted in response to different types of information signaling an objects’ CoM. They found shape and size to be good cues to the CoM, whereas a verbal instruction or an artificial visual cue (colored dot) is less effective. However, it is difficult to explain why materials should be an effective cue to the overall mass (Buckingham et al. 2009) but not to mass distribution. In this regard, it is interesting to note that a study by Crajé et al. (2013) found that participants could not adjust the initial torque based on visual information about density. Finally, we cannot exclude the possibility that the measures we used were not sensitive enough to capture the effects of expected material differences on the motor system.

### Experiment 2

#### Perception.

**Fig. 5.**
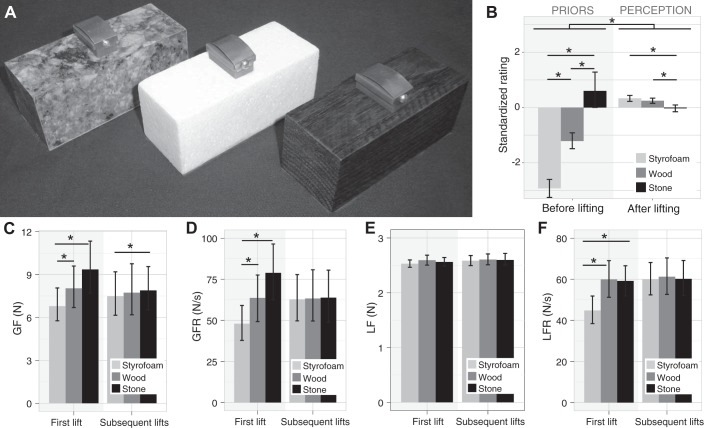
Stimuli and main results of *experiment 2*. *A*: the 3 objects used to test the classic material-weight illusion (MWI). All have the same mass, size, and shape but appear to be made of different materials (stone, Styrofoam, and wood). *B*: results of the perceptual rating. Bars on the *left* (shaded area) represent prior expectations, i.e., ratings before lifting; bars on the *right* represent reported heaviness, i.e., ratings after lifting. The *y*-axis shows mean ratings in *z*-scores; the lower the score the lighter the object appeared, and the higher the score the heavier it appeared. Bars show mean across participants; error bars, 95% confidence intervals. *C*: mean peak grip force (GF) for different materials in first and subsequent lifts. *D*: mean peak rate of change of GF for different materials in first and subsequent lifts. *E*: mean peak load force (LF) for different materials in first and subsequent lifts. *F*: average peak rate of change of LF for different materials in first and subsequent lifts. All error bars show 95% confidence intervals. GFR, grip force rate of change; LFR, load force rate of change.

We were able to replicate the classic MWI with the objects used in our experiment. Results of the perceptual rating are depicted in Fig. 5*B*. Before lifting the objects, participants expected the Styrofoam object to be lighter [−2.93 ± 0.17 (means ± SE)] than the wooden object (−1.22 ± 0.16) and the wooden object to be lighter than the stone object (0.60 ± 0.34; all *P* < 0.001; adjusted α = 0.0167). After they had lifted the objects, this pattern reversed; stone was on average perceived to be lighter (−0.03 ± 0.06) than Styrofoam (0.34 ± 0.06, *P* = 0.001) and wood (0.25 ± 0.05, *P* < 0.001). The difference between the latter two was not significant (*P* = 0.335). Besides the significant interaction between material and pre- versus post-lifting (for details, see Table 2), the ANOVA also revealed a significant main effect of the material and a significant main effect of lift. They were presumably driven by the fact that the expected differences between materials were much larger than the perceived differences reported after lifting. As in *experiment 1*, we calculated an illusion index for each participant. Results are shown in Fig. 3*D*. The majority of participants lie in the lower right quadrant; i.e., they experienced the classic MWI. A few participants lie on the horizontal axis; i.e., they did not experience an illusion, and one participant experienced an inverted MWI. A one-sample *t*-test showed that the illusion index after lifting was significantly smaller than zero [*t*(23) = −3.74, *P* = 0.001]. This figure also shows that the classic MWI seems to be smaller in size than the inverted MWI we found in bipartite objects. This observation was confirmed by a two-sample *t*-test that showed a significant difference [*t*(70.87) = 3.69, *P* < 0.001] between the absolute values of the illusion index in the two groups of participants (*experiment 1* vs. *experiment 1*).

**Table 2.** Results of the repeated-measures ANOVAs of experiment 2

Measure (Factor)	*df_1_*	*df_2_*	*F*	*P* Value
Heaviness rating				
Material	1.52	34.99	54.36	<0.001*
Lift	1	23	53.09	<0.001*
Material × Lift	1.36	31.26	75.32	<0.001*
Peak GF				
Material	2	46	9.58	<0.001*
Lift	1	23	0.51	0.484
Material × Lift	2	46	7.90	0.001*
Peak GFR				
Material	1.53	35.08	10.41	0.001*
Lift	1	23	0.00	0.969
Material × Lift	2	46	9.44	<0.001*
Peak LF				
Material	2	46	2.25	0.117
Lift	1	23	1.64	0.214
Material × Lift	2	46	0.82	0.447
Peak LFR				
Material	2	46	9.40	<0.001*
Lift	1	23	3.62	0.070
Material × Lift	2	46	8.12	<0.001*

GF, grip force; GFR, grip force rate of change; LF, load force; LFR, load force rate of change.

* Significant effects in the ANOVAs.

Taken together, the results of the perceptual ratings suggest that the findings of *experiment 1* cannot be explained by the specific shape, weight, or materials we used here. When appearing to be made of one uniform material (*experiment 1*), the same objects elicited the classic MWI, where heavier-looking materials (stone) are perceived to be lighter than lighter-looking materials (Styrofoam). This perceptual illusion was experienced by the majority of participants and lasted throughout the experiment. Thus, the inverted MWI in *experiment 1* is presumably related to the fact that the objects appeared bipartite.

#### Forces.

In accord with previous literature (Buckingham et al. 2009; Flanagan and Beltzner 2000), we analyzed the peaks of GF and LF and their rates of change to test whether they would be scaled to the expected weight in the first lift and then adjusted to the actual weight (i.e., no difference between materials) in all subsequent lifts. Such an effect could show up as an interaction between material and lift in the ANOVAs. This is indeed what we found for three of the four variables (all except LF; see Fig. 5,*C*–*F*, and Table 2). More specifically, for the peak GF (Fig. 5*C*), we found a significant main effect of material; GF was smaller overall for the Styrofoam object [7.14 ± 0.68 N (means ± SE)] than for the wooden (7.89 ± 0.83 N, *P* = 0.012) and stone objects (8.62 ± 0.82 N, *P* = 0.001). This difference was present in the first lift (Styrofoam vs. stone: *P* = 0.001; Styrofoam vs. wood: *P* = 0.010) and only for the stone-Styrofoam comparison also for later lifts [*P* = 0.010; all other *P* > 0.0167 ( = adjusted α)]; i.e., there was a significant interaction effect. There was no main effect of lift on the peak GF. A similar pattern was also observed for the peak rate of change of the GF (see Fig. 5*D*). We found an interaction between material and lift; the rate of change was lower for Styrofoam (47.96 ± 5.64 N/s) compared with stone (78.86 ± 8.83 N/s, *P* = 0.001) and compared with wood (63.64 ± 7.90 N/s, *P* = 0.007) in the first lift but not in later lifts [*P* > 0.0167 ( = adjusted α)]. Thus, the significant main effect of material was due only to the differences in the first lift. There was no main effect of lift. We found the same pattern of results for the peak LF rate, a main effect of material, and an interaction effect (see Fig. 5*F*); the rate of change was lower for Styrofoam (44.80 ± 3.67 N/s) compared with stone (60.16 ± 4.64 N/s , *P* = 0.002) and compared with wood (59.91 ± 4.77 N/s, *P* < 0.001) in the first lift, but not in later lifts [all *P* > 0.0167 ( = adjusted α)]. For the peak LF, we found no significant effect of material, lift, or their interaction, presumably because the variation was overall very small (see Fig. 5*E*).

Overall, we have replicated Buckingham et al. (2009), showing that the perceptual illusion appears to be dissociated from the forces applied when the objects are lifted. Initial forces in the first trial are scaled to the expected weight of the object based on prior assumptions about material properties; i.e., more force is applied faster to objects that appear to be heavier (stone) than to ones that appear lighter (Styrofoam). After the first trial, forces are adjusted to the actual mass of the object, which was the same for all materials; i.e., there were no differences between materials in the later trials. There were two exceptions. We did not find an effect for the LF [nor did Buckingham et al. (2009); this measure might simply not be sensitive], and we found a difference between the peak GF for Styrofoam and stone objects not only for the first but also later lifts. This is surprising, given that the actual mass of the object was exactly the same.

### Experiment 3

#### Perception.

**Fig. 6.**
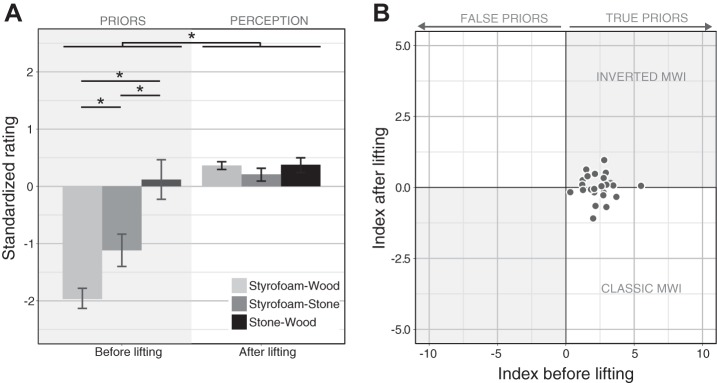
Results of *experiment 3*. *A*: standardized ratings averaged for each object across all participants and trials before (shaded area) and after lifting. *Significant differences between the perceived heaviness of the objects as well as between the perceived heaviness before and after lifting. Error bars show 95% confidence interval between participants. *B*: illusion index before vs. after lifting (perceived heaviness of stone-wood object − perceived heaviness of wood-Styrofoam object) for each participant in *experiment 3*. The axes are scaled as in Fig. 3 to facilitate comparison. Note, however, that here we compare the heaviest- to the lightest- looking object, whereas in Fig. 3 the index is based on comparing the heaviest- to the lightest- looking material. As in Fig. 3, participants in the *top right* (and* bottom left*) quadrant experienced the inverted material-weight illusion (MWI) (gray fields), whereas participants who experienced the classic MWI (white fields) fall in the *bottom right* (and *top left*) quadrant.

The expectations of the participants were in line with what we found in *experiments 1* and *2* (see Fig. 6*A*). The stone-wood object was expected to be heavier than the other two objects, and the Styrofoam-stone object was expected to be heavier than the Styrofoam-wood object. Differences between all objects were significant before lifting (all *P* < 0.001, adjusted α = 0.0083). These large differences were also responsible for a main effect of object in the repeated-measures ANOVA [*F*_(2, 46)_ = 62.64, *P* > .001]. After the objects were lifted they were rated heavier overall [main effect of lift: *F*_(1, 23)_ = 70.43, *P* < 0.001]. In addition to the main effects, we also found a significant interaction between the factors object × lift [*F*_(2, 46)_ = 53.73, *P* < 0.001]. After participants had lifted the objects, they were not perceived as varying in weight [all *P* > 0.0083 ( = adjusted α)]. Thus, when bipartite-looking objects are lifted (like in *experiment 1*) but the overall weight of the objects is rated (like in *experiment 2*), participants experienced no weight illusion, neither the classic nor the inverted MWI. Figure 6*B* shows the illusion index before and after lifting for each participant. Most participants lay on the horizontal axis, i.e., they experienced no illusion, whereas some individuals experienced an inverted MWI (Fig. 6*B*; *top* *right*) or classic MWI (Fig. 6*B*; *bottom right*). A one-sample *t*-test confirmed that on average the illusion index after lifting was not significantly different from zero [*t*(23) = 0.15, *P* = 0.89]. We conducted an additional Bayesian one-sample *t*-test using JASP (JASP Team 2018) to confirm this null effect. Indeed, we found that the data are 4.61 times more likely under the null hypothesis (BF_01_ = 4.614). The illusion index was significantly different from the illusion index in *experiments 1* [*t*(71) = 5.13, *P* > 0.001, adjusted α = 0.0167] and *2* [*t*(46) = −2.83, *P* = 0.007]. This result is very interesting because the same objects led to a strong weight illusion in *experiment 1*. The only difference between the two experiments was that here, instead of judging the mass distribution, participants had to judge mass. Remarkably, this same task of judging mass, on the other hand, also led to a weight illusion in *experiment 2*, but in the opposite direction. It almost appears as if the two illusions canceled each other in *experiment 3*, resulting in an average of no illusion. It might also be that separate mechanisms are responsible for the diverging effects in the three experiments or that there is a fundamental difficulty in integrating multiple weight or density estimates within a given object. Whatever the cause of the discrepancy of results, they suggest that the classic MWI diminishes in bipartite objects and that the inverted MWI seems to be related to judgments of mass distribution.

### Experiment 4

#### Perception.

**Fig. 7.**
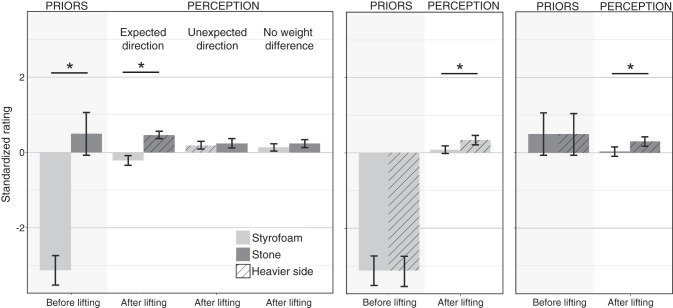
Perceptual results of *experiment 4*. Perceptual ratings of the halves of each object before and after the 3 bipartite-looking objects are lifted (*left*) and the 2 uniform-looking objects (*right*). Results of the perceptual rating. Bars in the shaded areas represent prior expectations, i.e., ratings before lifting; bars in the unshaded areas represent reported heaviness, i.e., ratings after lifting. The *y*-axis shows mean ratings in *z*-scores; the lower the score the lighter the object appeared, and the higher the score the heavier it appeared. Bars show mean across participants; error bars, 95% confidence intervals. *Significant difference between the ratings of both halves of the object.

As in *experiment 1*, participants expected the Styrofoam half to be significantly lighter [−3.13 ± 0.21 (means ± SE)] than the stone half [0.50 ± 0.30; *t*(24) =−9.01, *P* < 0.001] in bipartite-looking objects (see Fig. 7). In contrast, participants did not expect a difference between the halves of uniform-looking objects (see Fig. 7). Because there was no difference in the ratings of any individual participant, we did not calculate the statistics on the group level for this comparison. These results confirmed that the appearance of the objects induce the expectations we intended.

Central to our research questions were the heaviness ratings after lifting the objects on each trial. For bipartite objects with a weight difference in the expected direction, i.e., stone heavier than Styrofoam, participants also perceived the stone half to be significantly heavier (0.46 ± 0.05) than the Styrofoam half [−0.22 ± 0.07; *t*(24) = −7.102, *P* < 0.001; see Fig. 7). However, if the weight difference was in the unexpected direction, i.e., Styrofoam was physically heavier than stone, both halves were perceptually equal [*t*(24) = −0.72, *P* = 0.476]. Thus, making the Styrofoam half 100 g heavier than the stone half seemed to cancel out the inverted MWI that we observed in *experiment 1*; heavy Styrofoam was perceived as heavy (0.19 ± 0.05) as light stone (0.24 ± 0.06). This is similar to an experiment by Buckingham et al. (2009) in which making the heavier-looking object physically heavier (720 g) than the lighter-looking object (680 g) canceled out the classic MWI. Interestingly, in our experiment the perceptual difference between two identically weighted halves of a bipartite object was smaller than could be expected based on the results of *experiment 1* and did not reach significance [*t*(24) = −1.637, *P* = 0.115]. Styrofoam was perceived not to be significantly lighter (0.14 ± 0.05) than stone (0.24 ± 0.06). This indicates that not only the weights of the two halves of the object lifted in a given trial but also the weight of the comparison objects lifted in previous trials were integrated into the heaviness percept. Participants reported a perceptual difference within the uniform-looking objects after lifting. For both objects, the physically heavier side was also perceived to be heavier (stone: 0.31 ± 0.07; Styrofoam: 0.35 ± 0.07) than the physically lighter side [stone: 0.03 ± 0.06, *t*(24) = −3.43, *P* = 0.002; Styrofoam: 0.09 ± 0.05, *t*(24) = −3.43, *P* = 0.002]. When comparing the perceived weight difference in the uniform-looking objects to the object with the expected difference in paired *t*-tests, we found that the expected weight difference was significantly larger than the unexpected weight difference (both *P* < 0.001, adjusted α = 0.025).

In summary, we found the largest perceptual difference when participants expected a difference, i.e., in bipartite objects with a heavy stone and a light Styrofoam half. Smaller but significant weight differences were perceived in uniform-looking objects, for which participants did not expect a weight difference. However, when a weight difference was expected (i.e., bipartite appearance) but was either absent or in the opposite direction, participants did not perceive a weight a difference. Our results are in support of the theory that weight perception is an integrative process in which prior expectations and incoming sensory information from lifting the target object, as well as an anchor from the comparison objects lifted in the previous trials, are integrated into a weight percept. Other studies show evidence that the perceived weight of an object is modulated by the weight of the object lifted in the previous trial (Maiello et al. 2018; van Polanen and Davare 2015). This might also be true for the perception of different weight distributions in consecutive trials, where the overall weight is constant as in our experiment. Such trial effects are likely the explanation for why we do not find an inverted MWI for the bipartite object with uniform weight distribution. However, we cannot exclude the possibility that we would have found an effect with a larger sample size (although the same sample size had sufficient power in the first 2 experiments).

#### Torque.

**Fig. 8.**
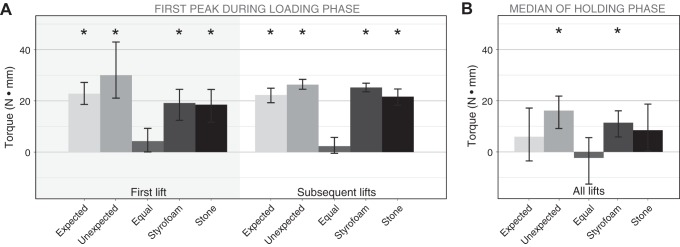
Torque measurements. *A*: mean initial peak torque around the *y*-axis during the loading phase in first and subsequent lifts. An initial rotation toward the heavier side is indicated by positive values; negative values indicate a rotation toward the lighter side. No rotation would result in a torque of zero. In the case of the object with equally weighted halves, positive torque values indicate a rotation toward the heavier-looking side. Error bars show 95% confidence intervals. *Average value to be significantly different from zero. *B*: mean of the median torque around the *y*-axis during the holding phase of the movements in all lifts; same notation as in *A*.

The initial peak torque during the loading phase was completely driven by the weight differences between the two object halves in the first as well as all later trials (see Fig. 8*A*). Each object was initially tilted toward its heavier side (all *p* values < 0.001, adjusted α = 0.005). Only the bipartite-looking object with a uniform mass distribution showed no significant torque in the first [*t*(24) = 1.74, *P* = 0.095] or later lifts [*t*(24) = 1.36, *P* = 0.187]. Visually, all three bipartite objects have the same appearance, but there was either no torque, torque in the direction of the Styrofoam half, or torque in the direction of the stone half. Thus, the visual appearance had no influence on the initial torque. Results were the same when considering only the first bipartite-looking object that each participant lifted for the analysis. Whether this was due to the fact that the appearance of our objects was not indicative of their weight distribution or whether participants were more generally unable to counteract an uneven mass distribution in an anticipatory fashion is not clear from our data. Results from Crajé et al. (2013) suggest that participants can learn to adjust their grasp to reduce the initial tilt of objects with nonuniform density within few trials.

After the initial torque toward the heavier side of the objects, participants corrected their movement and reduced the torque during the holding phase of the movement (see Fig. 8*B*). Only for the object that appeared to be completely made of Styrofoam and the bipartite object with the unexpected weight distribution was there still a significant torque toward the heavier side [Styrofoam: *t*(24) = 4.10, *P* < 0.001; Unexpected: *t*(24) = 4.64, *P* < 0.001; all other *P* values < 0.01 ( = adjusted α)]. This indicates that after the initial error signal, participants were able to adjust their grip to counteract the nonuniform density at least partly. Because we did not measure object tilt directly, however, we cannot say how strongly the objects were tilted during the holding phase.

## DISCUSSION

The main finding of this study is that the violation of an expected weight distribution leads to a novel weight illusion. In *experiment 1*, we found that in bipartite objects, for which one half looks significantly heavier than the other half, the heavier-looking side is perceived to be heavier when lifted, although the true mass of both sides is the same. This effect was robust over the whole duration of the experiment, in a large group of participants, and across two different perceptual judgments. Strikingly, this illusory effect in the opposite direction of the well-known MWI, in which equally weighted but heavier-looking objects feel lighter. *Experiment 2* ruled out the possibility that this inversion of the MWI was due to any other object property of our stimuli than their being bipartite. We replicated the classic MWI for uniform objects of the same size, weight, and materials as in *experiment 1*. When combining the bipartite stimuli of *experiment 1* with the perceptual task of *experiment 2* (estimating weight of entire objects), in *experiment 3* we found that no illusion was perceived (i.e., neither the classic nor the inverted MWI). Finally, in *experiment 4*, we tested whether prior expectations are integrated (as suggested by *experiment 1*, where the perceived weight lies in between prior and sensory estimate) or contrasted (as suggested by *experiment 2*, in which the perceived weight lies outside the range between prior and sensory estimate and in opposite direction of the prior) with sensory information if objects have a nonuniform weight distribution. Interestingly, and consistent with an integrative process, we found that the same weight difference of 100 g between the halves of an object can subjectively feel absent, small, or large, depending on the prior expectations of the weight distribution. A discrepancy between expected and actual weight distribution in opposite directions induced the illusion of a uniform weight distribution. In other words, making the lighter-looking side of a bipartite object 100 g heavier cancelled out the inverted MWI so that both sides felt equally heavy. If the discrepancy between expected and actual weight distribution was smaller, i.e., when a uniform distribution was expected, the perceived difference between the sides was small. If, on the other hand, there was no discrepancy between expected and actual weight distribution (or at least both were in the same direction), the same 100-g difference was perceived to be very large. In comparison to the four objects with a nonuniform weight difference, a bipartite-looking object with equally weighted halves was not perceived to differ in weight (unlike *experiment 1*). This suggests that the weight distributions of reference objects experienced in the same context also affect subjective ratings, presumably by anchoring the range of the rating scale. In *experiments 1* and *4*, the scale was presumably anchored to the visual ratings as well as the weight of the wooden object used in the practice trials, which would predict no difference between the scales. In *experiment 4*, however, the rating scale may have additionally been anchored to the weight differences in the other stimuli. Specifically, although the absolute sensory reliability of the “no weight difference” judgment should be the same in both *experiments 1* and *4*, in the context that includes large real weight differences (i.e., *experiment 4*), the relative size of the sensory uncertainty distribution would be small compared with the total range of sensory signals experienced across objects. In contrast, when the same no weight difference judgment is compared across a set of objects all without any weight difference (as in *experiment 1*), the relative size of the uncertainty distribution of the no weight difference judgment would be large compared with the range of experienced sensory signals. When combined with the same prior, the narrower sensory estimate (in *experiment 4*) should lead to an overall estimate that is shifted further toward no weight difference.

The main question that arises from our results is why seemingly similar tasks (estimating weight in bipartite versus uniform objects) lead to opposing perceptual estimates: the inverted MWI, the classic MWI, or no illusion (as in *experiment 3*). Our results challenge existing theories of weight illusions. Not unexpectedly, the findings speak against the sensorimotor mismatch hypothesis; i.e., we did not find any systematic coupling between perception and action. Instead, for uniform-looking objects we replicated earlier findings (MWI: Buckingham et al. 2009; SWI: Flanagan and Beltzner 2000) that forces are tuned to the expected weight of the objects in the first trials and then adjusted to the actual mass, although the perceptual illusion persists. In case of bipartite objects, we found no effect in the first or later trials. Taken together, these findings do not support the sensorimotor mismatch hypothesis but instead suggest that the perceptual illusion is independent of the motor system. Results from *experiment 4* suggest that even on the first lift the grip is not scaled to counteract an anticipated torque; instead, a torque emerges (in case of an uneven mass distribution) and is then corrected. Presumably, participants followed the same strategy in *experiment 1*, with the only difference being that there was no torque signal to counteract. This might explain why we did not find the expected effect on the motor system in *experiment 1*. We cannot exclude the possibility that there was an effect, but our measures were not sensitive enough to capture it.

The classic MWI is often explained with a perceptual contrast resulting from the violation of expectations; e.g., a Styrofoam object is heavier than expected and thus feels even heavier than the same object with a stone appearance. If the expectations for bipartite objects are weaker than for uniform objects, one may expect to find the MWI to disappear, like we found in *experiment 3*. However, the same violation of expectations was present in *experiment 1*, yet this led to a percept shifted in the opposite direction of the classic illusion. Therefore, violation of expectations alone cannot explain the occurrence and direction of the classic and inverted MWI. Refining this theory by differentiating between violations of expectations about weight and expectations about a weight distribution may formally close that gap, but such an account lacks explanatory depth, as it remains unresolved as to why there should be differences between the two. It might be that expectations are stronger in one case than in the other (weight vs. weight distribution) or that the violation is stronger in one case. We do not see evidence for either in our data, and it is questionable how such a theory would account for the outcomes of all four experiments. However, a more systematic test of exactly that question is required. The classic MWI has been suggested to be an “anti-Bayesian” mechanism that marks outliers in the environment (Baugh et al. 2012). This idea would need to be refined for it to be able to explain why the anti-Bayesian mechanism does not apply in the case of weight distribution outliers. For example, it might be the case that the distribution of weights in the environment is much narrower than the distribution of weight distributions (or CoM positions); therefore, the experimentally modified uniform stimuli of the classic MWI fall far outside that range and will be marked as outliers, whereas the bipartite stimuli fall within the broad distribution and will be integrated with the prior. However, it is unclear why the bipartite objects would neither be marked as an outlier nor be integrated with the prior in case of weight judgments. Future studies should aim to test this refined theory.

In summary, potential explanations of the classic MWI in their current form fail to explain the inverted MWI in bipartite objects, as found in *experiment 1*. At the same time, the standard Bayesian integration framework can presumably account well for the inverted MWI in bipartite objects and the results of *experiment 4* (although we did not test this idea specifically) but fails to explain the classic MWI in uniform objects.

However, a modification of the standard Bayesian framework has been shown to successfully predict the related SWI. Peters et al. (2016) proposed a model that predicts the illusion as the result of Bayesian integration in a framework of multiple competing density priors (as proposed by Yuille and Bülthoff 1996) and the likelihood of incoming haptic information. These same authors recently proposed a similar mechanism underlying the classic MWI (Peters et al. 2018). Within this framework, the classic and inverted MWI may reflect two different estimates resulting from the same basic mechanism. Specifically, under normal circumstances and the assumption of uniform density, there is a strong relationship between a material’s appearance and its weight, leading to a strong expectation that stone is heavier than Styrofoam by a specific amount. However, we might also experience a significant number of counterexamples such as objects that mimic a certain material, e.g., light objects with a fake-stone veneer, or objects covered with a different material, e.g., heavy objects covered in Styrofoam to protect them during transportation. Such alternative relationships between material appearance and weight could have distinct “atypical” priors, each representing competing expectations about the density relationships. Each of the competing expectations has an individual a priori probability and hence, results in a different likelihood of the incoming sensory information. As a result, there would be multiple competing posterior probabilities (1 for each expected density relationship), of which the maximum will be selected to produce a final weight estimate within the competitive prior framework. This is fundamentally different from the standard Bayesian explanation in which only one prior (“stone is heavier than Styrofoam”) modifies the likelihood of the incoming sensory information and results in just one posterior probability. Only Bayesian integration of the likelihood of incoming sensory information given competing expectations and their prior probabilities can result in a percept shifted toward an a priori unlikely expectation, as Peters and colleagues (2016) have shown for the SWI.

Applied to our study, we may assume the same a priori probabilities of the different density relationships, because the expectations about materials were the same no matter whether the objects were bipartite or uniform. The fundamental difference between the two experiments was the type of sensory estimate required to make the perceptual judgment: an estimate of mass or an estimate of mass distribution. Although both mass and its first moment (distribution) contribute to the perception of weight, their sensory estimates may differ in reliability. For example, it may be that the haptic estimate of mass is more reliable than the haptic estimate of its distribution or vice versa. Therefore, our second assumption is that the sensory estimates of mass and mass distribution vary. Importantly, this refers to the reliability of the estimate by the sensorimotor system; therefore, it is unrelated to the force and torque measurements we took. Although both sensory estimates may influence perception in *experiments 1* and *2*, it is likely that their influence varies, depending on the task. The sensory estimate of mass distribution presumably has greater influence when object parts are judged in *experiment 1*. Given the same competing prior expectations (*assumption 1*) but differences in the reliability of the incoming sensory information (*assumption 2*), the likelihood of the sensory information will vary between *experiment 1* and *experiment 2*. Thus, the same Bayesian integration mechanism could result in different final weight estimates; it could be shifted toward the more likely a priori expectation that stone is heavier than Styrofoam in one case (*experiment 1*) and shifted toward the opposite (and a priori less probable) expectation that Styrofoam is heavier in *experiment 2*. A final weight estimate that falls somewhere between the opposing percepts could result if the relative influence of the two sensory estimates changes. This could happen, for example, when participants are asked to judge the weight of entire objects that appear to have a nonuniform weight distribution, as in *experiment 3*. In this case, the sensory estimate of mass distribution might have a larger influence than when the weight of uniform objects is judged. The results of *experiment 3* are in line with this idea.

An integration mechanism is in line with previous literature (Adams et al. 2004; Ernst and Banks 2002; Ernst and Bülthoff 2004; Kersten and Yuille 2003; Körding et al. 2004; Körding and Wolpert 2004; Langer and Bülthoff 2001; Sun and Perona 1998; Weiss et al. 2002) and in agreement with our data from the four experiments presented here. However, because this model is only a post hoc explanation of our results, future studies should test it systematically. If the Bayesian account proposed by Peters et al. (2016) can explain the SWI (Peters et al. 2016), the classic MWI (*experiment 2* and Peters at al. 2018), the inverted MWI (*experiment 1*), the absence of an illusion (*experiment 3*), and weight perception in objects with a nonuniform weight distribution (*experiment 4*), one might also expect to find an inverted SWI in bipartite objects with unequally sized halves but equal weight distribution. Although it is technically challenging to produce objects that have a different volume but the same rotational momentum and the same mass in each half, this would be a powerful test of a shared underlying process. If there is an inverted SWI in bipartite objects, this would speak in favor of a common mechanism underlying different weight illusions and will potentially provide insights into weight perception in general.

Although only behavior was measured in this study, one can speculate about the neurobiological mechanisms underlying the findings. To make the visual judgement before the first lifting trial, prior knowledge about material classes and their associated properties needs to be activated. Classification of materials and their properties progresses along the ventral visual stream (Cant and Goodale 2007, 2009; Cavina-Pratesi et al. 2010a, 2010b; Hiramatsu et al. 2011). When the object is lifted, this visual information about materials needs to be transformed into motor commands. A whole network of brain areas is involved in even a simple two-finger grip to lift and hold an object as in our experiments. Gallivan et al. (2014) identified brain areas from whose activation pattern the texture and/or weight of an object can be successfully decoded during or before lifting the object. Their results suggest that premotor and primary motor cortex encode weight during planning and execution of lifting movements, whereas the somatosensory cortex represents weight information only after an object is touched. Interestingly, if the weight of an object could reliably be derived from its visual texture (either through knowledge about materials or associations between an object and its weight learned during the experiment), then ventral texture-sensitive regions appeared to code information about the weight of the object. Thus, it seems likely that both dorsal and ventral visual networks are involved in the visuomotor transformations that anticipate the forces required to lift a heavy or light object. In our study, we found strong evidence that grip and load forces were scaled according to prior knowledge or sensorimotor memories in *experiment 2*. However, there may be differences in how well the forces can be adjusted to the overall weight or the distribution of weight. If the forces are not sufficiently adjusted a priori or such adjustment is not possible, e.g., because the texture is uninformative about the weight (as in *experiment 4*), grip force will be corrected online through cutaneous feedback. Such correction is very fast (<100 ms; e.g., see Johansson and Westling 1984) and presumably highly automatic, although the underlying neural mechanisms are not yet well understood (for a review, see Johansson and Flanagan 2009). Future research is required to better understand the underlying neurobiology.

## GRANTS

This work was supported by the DFG International Research Training Group IRTG 1901 “The Brain in Action—BrainAct,” the DFG SFB-TRR 135 “Cardinal Mechanisms of Perception,” and the European Research Council Consolidator award “SHAPE” (ERC-CoG-2015-682859).

## DISCLOSURES

No conflicts of interest, financial or otherwise, are declared by the authors.

## AUTHOR CONTRIBUTIONS

V.C.P., G.B., M.A.G., and R.W.F. conceived and designed research; V.C.P. performed experiments; V.C.P. and G.B. analyzed data; V.C.P., G.B., M.A.G., and R.W.F. interpreted results of experiments; V.C.P. prepared figures; V.C.P. drafted manuscript; V.C.P., G.B., M.A.G., and R.W.F. edited and revised manuscript; V.C.P., G.B., M.A.G., and R.W.F. approved final version of manuscript.
